# Heart Failure with Preserved Left Ventricular Ejection Fraction: A Complex Conundrum Simply Not Limited to Diastolic Dysfunction

**DOI:** 10.1155/2023/1552826

**Published:** 2023-07-18

**Authors:** Angel Lopez-Candales, Talal Asif, Khalid Sawalha, Nicholas B. Norgard

**Affiliations:** University of Missouri-Kansas City, School of Medicine, Kansas City, Missouri, USA

## Abstract

Over the last two decades, the changing paradigm of heart failure with preserved ejection fraction (HFpEF) has transformed our understanding not only of the pathophysiology of this clinical entity but also the diagnostic and therapeutic approaches aimed at treating this complex patient population. No longer HFpEF should be seen as simply left ventricular diastolic dysfunction but as a group of that in addition of having small and thick left ventricles with abnormal diastolic filling patterns as their main pathophysiologic abnormality; they also have whole host of different abnormalities. In fact, this heterogeneous clinical entity embodies numerous mechanisms and is linked to multiorgan dysfunction, with hypertension and obesity playing a major role. Although we have gained an enormous amount of understanding not only on the causes but also the downstream effects of HFpEF, there is still much to be learned before we can fully comprehend this complex clinical entity. It is the main intention of this review to synthesize the most recent attributes, mechanism, diagnostic tools, and most useful therapeutic alternatives to be considered when evaluating patients either complaining of dyspnea on exertion as well as exercise intolerance or those recently admitted with HF symptoms but with normal LVEF in the absence of any other valvular abnormalities

## 1. Introduction

Over the last 3 decades, heart failure (HF) has become an increasingly important public health problem. As reported on the recent 2022 American Heart Association, the lifetime risk of developing HF remains high, with variation across racial and ethnic groups ranging from 20% to 45% after the age of 45 [1, 2].

While definitions used to differentiate HF with preserved ejection fraction (HFpEF) from HF with reduced (HFrEF) have been somewhat fluid over time and there is a wide data heterogeneity, recent estimates project that HFpEF will become the dominant form of HF while HFrEF will show a decreasing prevalence trend [3].

While adoption of the new universal HF definition eliminated the previously used ambiguous approach to characterize and treat HF, standardization of terms and the use of a more focused approach not only has narrowed the communication gap between clinicians and researchers but also empowered healthcare providers and institutions to better serve patients by delivering more effective health care services [4–14].

As our understanding of HFpEF continues to improve, it has become apparent that fundamental differences exist between HFrEF and HFpEF that could not be simply explained based on difference in EF values. In fact, current data seem to point out that numerous abnormalities are particularly relevant to HFpEF patients. The latter might not only explain differences in terms of clinical presentation and overall outcomes but also diagnostic considerations as well as therapeutic management.

**Figure 1:**
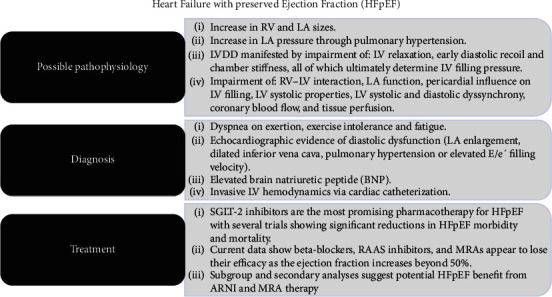
Graphical abstract illustrating the concepts defined in the review. RV: right ventricle. LA: left atrium. LVDD: left ventricular diastolic dysfunction. LV: left ventricular. SGLT-2: sodium-glucose cotransport-2. RAAS: renin-angiotensin-aldosterone system. MRA: mineralocorticoid receptor antagonist. ARNI: angiotensin receptor neprilysin inhibitor.

This review will revisit our current pathophysiologic understanding of HFpEF and how the different phenotypic expression of this clinical entity can be characterized and diagnosed while providing an updated version of the latest therapeutic options that are now clinically available (Figure 1).

### 1.1. Pathophysiology

First order of business is to recognize the distinction that exists in terms of LV diastolic dysfunction (LVDD) and HFpEF. Specifically, even when LV LVDD plays a critical role in the pathophysiology of HFpEF [15], not all patients with LVDD have or will develop clinical HFpEF [16, 17]. In contrast, all patients with HFpEF will have LVDD [16, 17].

To better understand the importance of LV diastolic function in terms of cardiac performance, it is critically important to recognize the specific mechanical elements responsible to determine LV diastolic function including LV relaxation, chamber stiffness, and early diastolic recoil, all of which ultimately determine LV filling pressure [18].

Equally relevant is to keep in mind that LV diastolic function is not simply dependent on LV of these mechanical properties; but it is also modulated by a series of other mechanisms that include right ventricular–LV interaction, left atrial function, pericardial influence on LV filling, LV systolic properties, LV systolic and diastolic dyssynchrony, coronary blood flow, and tissue perfusion [19].

In its most simplistic form, diastole can be divided into 4 components that include (1) isovolumic relaxation, (2) early filling, (3) diastasis, and (4) atrial systole for those in normal sinus rhythm [20]. Furthermore, the amount of LV filling that occurs during each of these phases have been shown to be clearly dependent on myocardial relaxation; the passive characteristics of the LV; the characteristics of the left atrium, pulmonary veins, and mitral valve; and the heart rate [21]. With regards to heart rate, only diastasis is closely dependent by increases in heart rate while the duration of both E and A waves are minimally heart rate dependent [20].

Now, these 4 components must be seen in their proper perspective, and to accomplish this goal, it has been proposed to envision the heart as a muscle-powered oscillator. When seen in isolation, the LV is a single woven muscle group that has no opposing muscle group; however, the oscillatory function is dependent on the systolic (contraction) and diastolic relation function of the LV. Consequently, the LV contracts in systole and expands in early diastole in accord to the well-known extracellular and intracellular elastic elements that include the extracellular matrix primarily made of collagen and elastin, the intracellular giant protein titin, and the visceral pericardium, which acts like a “shrink-wrap” and stores elastic energy in systole, contributing to the elastic recoil in diastole [22–24].

The almost perfect harmony existing between LV systole and diastole must be mechanically coupled so that the functions of both cycles are preserved. Explicitly, elastic energy is stored during LV contraction, which powers the chamber wall to recoil or to continue with unopposed relaxation as sarcomere cross-bridge uncoupling proceeds so that both chamber properties of stiffness and relaxation simultaneously determine global diastolic function [25]. Consequently, it is quite evident that LV diastolic function depends on the load. Specifically, the volume ejected affects end-systolic strain and the storage of elastic potential energy in tissue [25]. Therefore, the early phase of LV diastole, the stored elastic strain energy, from the previous systolic cycle, is released upon muscle relaxation [25]. The recoil generated by the LV elastic elements allows LV filling until the LV is fully relaxed, and equilibrium occurs during diastasis [25].

Furthermore, it is critically important to recognize changes in left atrial (LA) size although quite complex and multifactorial [26]; these changes become relevant in terms of LV diastole. Not the LA contractility is crucial to complete normal LV filling in patients with normal sinus rhythm, but also, LA volumetric changes reduce hydraulic force [26]. The latter prevents the LV from fully expanding longitudinally, and consequently, this would ultimately impair LV diastolic filling [26].

In so far, it is quite apparent that aside from intrinsic abnormalities within the LV myocardium that directly control chamber stiffness and relaxation, the volumetric load or preload as well as effective atrial contraction is all needed to accomplish normal LV filling.

Another important element, closely related to changes in LA size has been described by Kovacs' group as a piston function to characterize the relationship between the LV and LA during the cardiac cycle [26]. This interaction between the LV and LA was depicted by the basal-to-apical motion of the mitral annulus (MA) during the cardiac cycle [26]. Careful assessment of this motion described the presence of parallel hydraulic and restoring forces generated by LV ventricular contraction [26]. While hydraulic forces aid in LV lengthening during diastole, facilitating displacement of the MA being a direct consequence of the diastolic blood chamber pressure acting upon the anatomic surfaces of the heart, restoring forces are mainly generated on a molecular level within the myocardium [26].

### 1.2. Diagnosis

After this brief pathophysiologic introduction describing the most important relevant elements describing LV diastole, it is time to describe ways to diagnose LVDD. Even though invasive LV hemodynamics via cardiac catheterization studies have been traditionally considered the gold standard approach allowing quantification of the rate of myocardial relaxation, echocardiography (echo) has now surpassed invasive methods as the preferred noninvasive imaging tool currently used for routine diagnosis and characterization of LV diastolic dysfunction [19, 21, 27]. Furthermore, echo not only helps grouping patients within the broad umbrella term of HFpEF into different phenotypic categories, but it also aids in identifying unique pathophysiological mechanisms that may guide specific therapies [28].

However, it is critically important to recognize that echo is not perfect, and certain limitations need to be reviewed. First, traditional echo-Doppler variables such as the E/A ratio, isovolumic relaxation time, deceleration time, and pulmonary vein Doppler do not allow direct measurement of LV relaxation, stiffness, or filling pressure [28–31]. Second, all these conventional echo-Doppler variables are more accurate in patients with HFrEF but not in HFpEF [30, 32]. Third, a weak correlation exists between isovolumic relaxation time and *τ* (tau) [31, 33].

Despite the relative simplicity of the 2016 publication by the American Society of Echocardiography and the European Association of Cardiovascular regarding their recommendations pertaining LV diastolic function assessment [28], not only do these guidelines remain somewhat complex regarding their day-to-day utility in diagnosing and grading LVDD, but also, a considerable number of patients cannot be accurately diagnosed or classified [34–36].

Despite these limitations, a complete echo examination to assess LV diastole should be basic information regarding age, gender, body surface area, heart rate and rhythm, and blood pressure.

Once this basic demographic data is obtained, quality of all echo and Doppler signals must be determined as this will ultimately ascertain the feasibility if each study in validating or eliminating the possibility that this imaging tool would not be able to establish a definitive diagnosis [28]. The 2016 guidelines clearly delineate the potential limitations of echo and Doppler variable as well as in which clinical conditions their diagnostic value might either be limited or nondiagnostic [28].

Once we have made these determinations, critically important variables that need to be acquired in each comprehensive study need to include measures of LA and LV volumes that are critically important as well as LV wall thickness, LVEF, presence of any valvular abnormalities, and severity of tricuspid regurgitation with an estimate of pulmonary artery pressures [28].

As in any other clinical case, the presence of a single measurement that falls within the normal range does not necessarily indicate normal LV diastolic function. That is, we should strive for consistency between two or more of the indices, and echo indices of LV diastolic function should always be interpreted in a wider context that includes clinical status and the other 2D and other Doppler parameters [28]. The latter becomes particularly relevant when trying to apply any of these variables and interpret them between normal and abnormal LVEF or in patients with certain types of cardiomyopathy or rhythm abnormalities [28].

Even when on this writing, we would not comment on all potential echo Doppler measurements recommended by the American Society of Echocardiography for LV diastolic function assessment [28]; we would like to highlight the utility of tissue Doppler imaging (TDI) as it relates to MA diastolic velocities.

Even though introduction of this tool has certainly advanced our understanding and provided a more accurate as well as reliable characterization of LV diastolic function, there is a well-described difference between medial and lateral MA diastolic velocities [28, 33, 37]. Although not adopted by everyone, most will attest to the fact that lateral MA velocities provide more reliable information with regards to LV relaxation and compliance indexes when compared to PV-loop analysis than the septal MA velocities [38].

Thus, in this review we will continue referring to the lateral MA in our discussion of LV diastole, specifically, to the early MA diastolic (e') TDI velocities. Not only this e' velocities have been identified as important prognosticator but also the ratio that is obtained using the transmitral to this e' TDI velocity (E/e') [39]. These measurements have been particularly useful in patients with hypertension, HF, and post myocardial infarction and in patients undergoing stress echocardiography for suspected coronary heart disease [39].

More importantly, of all available echo LV diastolic parameters that can be used for assessing LV diastolic function, the peak MA TDI e' velocity has the strongest impact on cardiac mortality among other TDI variables [40]. Furthermore, this MA e' velocity provides incremental predictive power when managing cardiac patients [41].

Even though this review will not discuss all echo Doppler variables, Table 1 lists some supplemental variables that can certainly be useful in certain patients.

### 1.3. Heart Failure in Patients with Normal Left Ventricular Ejection Fraction

Before discussing HFpEF further, it is important to mention a clinical entity where it has variously been labeled as diastolic heart failure or heart failure with preserved LV function to a more preferred term now of heart failure with normal ejection fraction (HFNEF) as almost half of patients with symptoms of heart failure are found to have a normal LV ejection fraction, and the systolic function is not entirely normal [42]. The change in terms resulted after recent studies suggest that the physiological abnormalities in these patients are not entirely related to diastole only, and the systolic function is not entirely preserved when other measures are used besides ejection fraction [42]. Differential diagnoses in such patients with shortness of breath and LVEF > 50% would include cardiac and noncardiac causes certainly. Of these cardiac causes are HFNEF, coronary and valvular heart diseases, restrictive cardiomyopathies, constrictive pericarditis, intracardiac shunt, and hypertrophic obstructive cardiomyopathy (HOCM) [43]. Potential non-cardiac causes include deconditioning, anemia, lung etiologies, obesity, and thyrotoxicosis [43]. Further research is indeed needed to help explain the pathophysiology of HFNEF patients further in order to establish therapeutic strategies.

### 1.4. Heart Failure with Preserved Ejection Fraction: The Clinical Entity

As previously mentioned, the causality behind HFpEF extends well beyond the simplistic identification of LVDD. As already established, although LVDD plays a fundamental, overarching role in the pathophysiology of HFpEF [15, 19, 38, 39], emerging data over the past decade has conclusively demonstrated that other abnormalities within the cardiovascular system need to contribute to the development of HFpEF.

Explicitly, a complex interplay of numerous cardiometabolic diseases such as diabetes, obesity, and hypertension as well as contributing factors within the heart, systemic vasculature, and peripheral tissues has been described [15].

Before we discuss the complex interaction of metabolic factors identified in the clinical expression, we will first succinctly discuss some individual cardiac and peripheral vascular known to affect LV diastole. Understanding of the latter elements would certainly be beneficial when describing the different clinical HFpEF phenotypes.

### 1.5. Subtle Impairment in Left Ventricular Systolic Function

Despite the connotation of “preserved” EF, data has been clear in describing abnormalities in terms of systolic performance such as midwall shortening, torsion and twist, and circumferential and longitudinal shortening using tissue Doppler or strain imaging [44–47].

In addition, these subtle impairments in LV systolic function noted at rest are further worsened during exercise and likely explain the development of dyspnea on exertion and reduced exercise capacity in patients with HFpEF [48].

### 1.6. Left Atrial Remodeling

The importance of LA remodeling has gained enormous momentum as significant pathophysiological, structural, and mechanical functional changes have been identified not only in response to heart failure, hypertension, cardiac valvular disease, diabetes mellitus, and obesity, but also, these changes often become maladaptive and responsible for worse clinical outcomes [49].

The occurrence of LA remodeling differs significantly between the two main HF phenotypes, namely, HFpEF and HFrEF. Specifically, patients with HFrEF exhibit significantly greater LA dilation and mechanical systolic dysfunction [50]. In contrast, much increased LA stiffness and pulsatility is seen in patients with HFpEF. The latter mechanisms are likely responsible for a greater prevalence of AF among patients with HFpEF [50].

Based on these results, any intervention aimed at restoring the LA mechanical function is expected to provide favorable effects on pulmonary vasculature and right heart hemodynamics [50]. Conversely, interventions that decrease atrial contractility or adversely affect LA compliance will undoubtedly elevate pulmonary pressures resulting in right ventricular dysfunction and consequently worse clinical outcomes in patients with HFpEF [50].

For example, the resultant LA wall scarring and reduction in LA volumes seen after repeated radiofrequency AF ablations have been linked with development of pulmonary hypertension [51], while interruption of the LA appendage has been associated with the development of atrial stiffness that compromises atrial performance [52]. Therefore, future use of any of these interventions (LA closure device implants and AF ablations) should raise concerns and be carefully evaluated in future trials as these interventions might adversely affect pulmonary vascular-right ventricular interactions in patients with HFpEF [51, 52].

Since the occurrence of AF is known to affect exercise capacity and adversely affect outcomes in HFpEF [53, 54], introduction of supervised exercise training programs in selected patients with chronic and stable patients with both HFpEF and AF not only have been shown to be safe but also resulted in substantial clinical benefits, objectively measured as improved aerobic exercise capacity and quality of life scores [55].

Even though, individual drug treatment interventions will be addressed later; we believe it is important to introduce a relatively new concept that suggests implementation of a more holistic approach when managing AF in HFpEF. This was introduced in the ABC (Atrial Fibrillation Better Care) pathway [56]. In essence, this approach proposes the following steps: (A) avoidance of stroke with the use of appropriate anticoagulants; (B) better management of symptoms using a patient-centered approach using either a rate or rhythm control interventions; and (C) cardiovascular and coexisting-condition risk management [56].

Therefore, based on currently available data, it appears as even though HFpEF and AF coexist in many patients and this number is only expected to increase given the continued aging of the population, more studies are needed that will close the gap regarding the close interaction existing between the LV and LA as well as their relationship with the pulmonary artery to right ventricular functional unit. Certainly, much more understanding is needed to better decipher the mechanisms that lead to worse clinical outcomes as well as which therapeutic interventions would be best suited to curtail these adverse events. Certainly, we patiently wait the results of future trials.

### 1.7. Abnormal RV-Pulmonary Artery Coupling

The importance of the RV-pulmonary artery unit and coupling of this system is critically important in determining RV systolic performance [57]. Therefore, now, it is critically relevant to understand the role of pulmonary pressures in HFpEF. Specifically, pulmonary hypertension has been reported in up to 80% of patients of HFpEF [58, 59]. The development of pulmonary hypertension in HFpEF not only would explain complaints of dyspnea and worse exercise capacity, but also, pulmonary hypertension would further impair LV preload that will blunt RV systolic reserve, ultimately worsening clinical outcomes [60, 61].

Therefore, it would be clinically relevant to objectively assess RV systolic dysfunction when pulmonary pressures are elevated; however, RV dysfunction among HFpEF patients could also occur without RV pressure overload [59]. The relevance of this RV-pulmonary artery unit has described an initial linear relationship between RV function in pulmonary hypertension [62–64]. This initial linear relationship then becomes exponential when assessing RV performance in pulmonary hypertension [65]. However, specific cases illustrating the presence of RV dysfunction without elevation in pulmonary pressures include the RV dysfunction that occur in the setting of atrial fibrillation and the abnormal RV function that only occurs during exercise, even when resting RV function is normal [66, 67].

Knowing the well-described cascade of RV anatomical and adaptive changes that occur in response to elevation in pulmonary pressures [62–65], therefore, based on the tight functional RV-pulmonary artery unit and its effects on LV diastole, deterioration in RV structure and function will undoubtedly occur over time in HFpEF and vice versa [68].

Therefore, it is time to introduce the following echo Doppler variables that should be included in defining LVDD, particularly if pulmonary hypertension is noted [69–75]:

- Tricuspid annular plane systolic excursion (TAPSE)
- Tricuspid annular systolic tissue velocity (s')
- RV fractional area change
- RV index of myocardial performance
- RV free-wall strain
- Measures of atrial strain
- The recently described TAPSE/PASP ratio that has been found useful in assessing RV-PA coupling and particularly useful in identifying HFpEF patients at greater risk of adverse clinical outcomes.

### 1.8. Systemic Vascular Stiffening

Under normal circumstances, the ascending aorta recoils during each cardiac cycle to facilitate early diastolic left ventricular filling [76]. In sharp contrast, aortic stiffening, or loss of distensibility, has been associated with symptoms in patients with HFpEF [77]. This arterial stiffening has been traditionally viewed as the hallmark of aging simply reflecting changes in the mechanical properties of the arterial wall caused, particularly driven by the combined disorganization and fragmentation of elastin as well as accumulation and cross-linking of collagen [78].

Patients with HFpEF demonstrate increased arterial stiffening that is well beyond what expected for either normal aging or the presence of accompanying hypertension [79]. These vascular abnormalities have been linked to increases in LV afterload that in turn impair LV early relaxation, alter contractile function, contribute to the development of LV hypertrophy, and has been associated with the subsequent development of clinical HF symptoms [80–82].

### 1.9. Coronary Microvascular Dysfunction

The coronary microcirculation encompasses prearterioles and arterioles (< 500 *μ*m and <200 *μ*m in diameter, respectively) as well as capillaries. Even though, these minute vessels cannot be currently imaged despite advances in our current technology; functional assessment of the microcirculation can be attained either by invasive measures of coronary flow reserve or fractional flow reserve or noninvasively using positron emission tomography [83, 84].

The microcirculation is critically important in regulating myocardial blood flow, particularly when myocardial oxygen extraction is almost maximal at rest (20-fold higher than that of skeletal muscle) [85]. Under normal conditions, increases in oxygen demand can only be met by increases in coronary blood flow [85].

Current data suggests that the coronary microvascular is critically related to the development of HFpEF. Metabolic comorbidities highly prevalent in HFpEF can induce a proinflammatory state. A systemic inflammatory state can provoke microvascular endothelial dysfunction and a related reduction in nitric oxide bioavailability leading to negative cardiac remodeling and myocardial dysfunction [86].

### 1.10. Pulmonary Factors

Even when patients with HFpEF are known to have elevated in left heart pressures, it remains unclear how this affects pulmonary gas transfer or its determinants at rest and during exercise. This was addressed by Hoeper et al. that showed that patients with HFpEF that exhibit exercise intolerance have altered pulmonary function and gas exchange both at rest and especially during exercise [87].

In a study by Obokata et al., these investigators reported reduction in DLCO greater than 45% and conferred a threefold increased risk of mortality in patients with pulmonary hypertension and HFpEF when compared to patients with a DLCO > 45% [88]. These abnormalities can potentially be therapeutic targets to improve exercise tolerance in patients [87].

### 1.11. Peripheral Factors

The following peripheral abnormalities have been reported among patients with HFpEF [15, 89–91]:

- Reduced capillary density
- Increased intramuscular fat content
- Microvascular endothelial dysfunction that blunts exercise induced peripheral vasodilatation and thus reducing perfusion
- Decreased ability to peripherally extract oxygen
- Abnormal pulsatile aortic loading during exercise
- Reduced venous capacity
- Baroreflex-mediated venoconstriction
- Redistribution of venous blood, mostly because of active vasoconstriction causing decreased splanchnic venous capacity
- Reduced venous return
- Excess venous blood volume.

Furthermore, anemia has been commonly seen among patients with HFpEF, particularly, in the setting of chronic kidney disease [92]. Even though anemia is strongly associated with worse morbidity and mortality and pharmacological treatment, in principle, it appears as a straightforward intervention; unfortunately, current data has shown inconsistent results [92].

### 1.12. Pericardial Restraint

The increases in chamber sizes, wall thickness, and increased epicardial fat deposition, especially in obese patients with HFpEF, have been identified as potential contributors of pericardial restraint [93]. Since the pericardial space does not increase proportionally to the increase in cardiac chamber sizes, consequently, this results in an enhanced RV-LV interaction [15]. This enhanced RV-LV interaction simply means that any changes in right heart pressures influence left heart pressures in parallel [15].

### 1.13. HFpEF Clinical Phenotypes

Following this mechanistic preamble, healthcare providers would certainly appreciate if better categorization of the rather large population of HFpEF could be grouped together according to their clinical presentations. Although such approach might be extremely simplistic to treat a rather complex clinical entity, Bianco et al. proposed a hemodynamic-based classification [94]. These investigators divided patients into type 1 HFpEF from hypertrophic cardiomyopathy, type 2 HFpEF from infiltrative cardiomyopathies, type 3 HFpEF from nonhypertrophic cardiomyopathy without significant cardiovascular disease, and type 4 HFpEF from one or more cardiovascular conditions such as CAD and HTN and that is the most encountered group [95].

Rosalia et al. similarly categorized patients with HFpEF into pathophysiological categories to help practitioners deliver more individualized therapies [95]. They divided patients into obese phenotype, ischemic HFpEF, and cardiometabolic HFpEF [95].

We will discuss these phenotypes in more detail and elucidate the role of noninvasive imaging in phenotyping. Given the lack of universal effective pharmacological and device-based solutions for HFpEF, these classifications can help carefully select appropriate unique therapy on a case-by-case basis, especially with emerging new device therapies [96].

Aside from what we have already established regarding HFpEF and LV diastole, up to 33% patients in HFpEF echocardiographic substudies have normal diastolic function, even in those patients with elevated natriuretic peptides [97–99].

In general, hypertension (HTN) represents the most common comorbidity in HFpEF patients and is implicated not only in its pathogenesis but also in prognosis [100, 101]. Furthermore, of all potential comorbidities, it is probably the one that encompasses most HFpEF patients [102]. In addition, HTN precedes HF occurrence in 75% of cases and carries a sixfold increase in HF risk as compared to nonhypertensive individuals [97, 98]. In HTN, adaptive remodeling occurs that could be either concentric or eccentric hypertrophy [99, 101]. Although most hypertensive patients are at high risk of developing concentric hypertrophy, a growing proportion of subjects display a concentric-to-eccentric progression eventually leading to LV dilation and systolic dysfunction [98, 99].

Although considerable morphological heterogeneity exists between increased LV wall thickness and LA dilation, both considered hallmarks of HFpEF [102]; up to 50% patients with HFpEF have normal LV mass despite having HTN [103].

However, it is important to be reminded that although concentric remodeling and concentric hypertrophy are common forms of LV remodeling in HFpEF, both eccentric hypertrophy and concentric hypertrophy not only display similar elevations in cardiac filling pressures but also share worse clinical outcomes [102].

In terms of LA size, from one-third to one-half of patients with HFpEF that have normal LA size [100, 103, 104], a third of patients with HTN without HF have LA enlargement [105].

Another important comorbidity related to HFpEF is aging. Increasing evidence not only points out to significant differences that exist between healthy and unhealthy aging but also associated systemic changes that can occur because of these differences [106–109]. However, in general, aging is associated with complex changes within the cardiovascular structure, as listed on Table 2.

All these age-related abnormalities have been shown to compromise the interaction between the heart and vasculature, as this ventricular-vascular unit becomes decoupled, particularly during exertion [110]. The latter leads to the development of significant symptoms that limit exercise because of worsening LVDD and arterial stiffening [111–113].

Obesity would represent the next significant comorbidity associated with HFpEF. Multiple studies have demonstrated the close association existing between body mass index (BMI) and HF incidence [114–116].

Specifically, more than 80% of patients with HFpEF are either overweight or obese with median/mean BMI of 31 kg/m^2^ (TOPCAT study) and over 35 kg/m^2^ (RELAX trial) [117, 118]. In fact, significant weight loss, particularly seen after bariatric surgery, has been shown not only with educed LV mass but, most importantly, improved diastolic function [119].

This relationship between BMI and HFpEF must be better clarified. It has become more apparent that measurements of waist circumference and waist-hip ratio should be preferred over BMI when evaluating patients with HFpEF and increased body weight [120, 121].

However, it was quite interesting to highlight the results from the Dallas Heart Study which showed that central adiposity was linked to concentric LVH while lower body obesity with eccentric LVH [122].

When taken together, African American women are the most common ethnic group, independent from body composition to a higher prevalence of HFpEF [123].

Postmenopausal women have a higher incidence of LV diastolic dysfunction, and HFpEF is more prevalent in women than that in men [124]. It is postulated that age-related decline in estrogen contributes to the development of HFpEF by causing myocardial hypertrophy and diastolic dysfunction [124]. Furthermore, the decline in estrogen levels following menopause produces adverse modulation of the renin-angiotensin-aldosterone system, increased inflammation, enhanced oxidative stress, and endothelial dysfunction, leading to increased susceptibility to the development of HFpEF [124]. With the development of newer versions of hormone replacement therapy with fewer adverse effects, the role of estrogen in HFpEF in women may present a future therapeutic target.

In addition to obesity, physical inactivity and low fitness levels have also been identified as risk factors for developing subclinical cardiac structural abnormalities that herald development of HFpEF [125]. Observational data suggests that low fitness levels impact LV diastolic function more than LV systolic function [126]. Sedentary individuals have also been shown to have decreased LV compliance and distensibility when compared to individuals who exercise [127]. Moreover, improved physical activity portends a better prognosis and long-term outcomes among patients with HFpEF [127]. This benefit is postulated to be derived from maintaining a healthy sarcomeric mass with exercise and decreased inflammation-mediated myocardial fibrosis from decreased circulating levels of C-reactive protein [127]. The approach of combined caloric restriction and exercise training appears to hold promise in terms of improving symptoms in patients with HFpEF [128]. However, long-term data on clinical outcomes and mortality is lacking [125].

The next major phenotype associated with HFpEF is coronary artery disease (CAD). Even when obstructive CAD has been one of the most important causes of HFrEF, the relative importance of CAD has gained attention, and it is now being recognized in up to 53% of HFpEF registries [12, 129, 130]. In contrast to what we have described between HFpEF and both HTN and obesity as it relates to ethnicity, CAD is seen most among Caucasians [131]. In this regard, not only exclusion of CAD in Caucasians should be routinely performed using coronary artery angiography; but also, it is important to be reminded that in up to 30% of HFpEF patients, noninvasive testing fails to detect the presence of CAD [131]. Finally, not only does the combination of HFpEF and CAD results in greater deterioration of LV function and a worse prognosis when compared to simply having HFpEF [132, 133]; but also, performing either surgical or percutaneous coronary revascularization is performed in HFpEF patients with symptomatic CAD clinical outcomes that are improved [131].

Table 3 lists additional clinical entities that could be extremely useful to further improve phenotypic characterization of patients with HFpEF.

### 1.14. Pharmacotherapy

The search for pharmacologic therapies that improve major CV outcomes in HFpEF has been generally discouraging. Most positive outcome trials evaluating pharmacologic therapies for HF enrolled patients using a reduced EF as key entry criteria. ACE inhibitors, angiotensin receptor blockers (ARBs), beta-blockers (BB), mineralocorticoid receptor antagonists (MRAs), and SGLT-2 (sodium-glucose cotransport-2) inhibitors have demonstrated robust reductions in mortality and morbidity in HFrEF [134]. The efficacy of these pharmacotherapies has been shown to be greatest at the lower end of the EF spectrum and extending into the mildly reduced EF range [135]. However, their benefit has not been shown to consistently extend beyond an EF of 50% likely due to the heterogeneity of HFpEF with various underlying etiologies and pathophysiological abnormalities [135, 136]. Without robust data to guide HFpEF pharmacotherapy, treatment has focused on management of risk factors, comorbidities, and the relief of symptoms due to volume overload with diuretics (Table 4).

#### 1.14.1. Beta-Blockers

The efficacy of BB therapy in HFpEF remains undetermined, and BB use in HFpEF is controversial. Yet, despite the lack of high-quality evidence for BB benefit in HFpEF, observational data from contemporary HFpEF cohorts reveal that BB are nevertheless used at an extremely high rate in this patient population [137]. The rational for this high rate of BB use may be explained by a variety of practitioner assumptions. For instance, catecholamine excess and neurohormonal activation are pathophysiologic characteristics shared across the HF spectrum and can be constrained by BB therapy [138–140]. BB may be beneficial in HFpEF via blood pressure lowering and a reduction in LV hypertrophy, improvement in echocardiographic indices of diastolic function, and by slowing heart rate and reducing myocardial oxygen demand [141, 142]. BB are also efficacious in the treatment of common HFpEF comorbidities such as hypertension, coronary artery disease, and atrial fibrillation. Yet, outcome data for BB in HFpEF are sparse and have produced conflicting results.

A recent meta-analysis investigating the efficacy of BB at different EF ranges found that the lower the EF, the higher the benefit of BB [137]. There was evidence of CV protection from BB seen in the HFrEF and HFmrEF populations, but HF patients with an EF > 50% did not see any benefit [137]. Similarly, a Cochrane Database analysis of 10 studies and over 3,000 patients found limited benefit from BB in HFpEF [143]. Conclusions drawn from the analysis were limited due to the low quality of the available evidence. While the initial results suggested a potential reduction in CV mortality, the benefit did not persist following a sensitivity analysis of the data.

Heart rate has emerged as a risk factor in HFpEF. However, the complex heterogeneity of the HFpEF disease state is reflected in the way differing heart rate irregularities can impact HFpEF patients and alter the effect of BB therapy. Data show a high resting heart rate (≥70 beats per minute) that is a risk factor for adverse outcomes in patients with HFpEF [144, 145]. In this high-risk subgroup of HFpEF patients, data from the OPTIMIZE-HF registry suggest that BB therapy at high doses may lower the risk of all-cause mortality and the combined endpoint of all-cause readmission or all-cause mortality [146]. Conversely, chronotropic incompetence, defined as the inability of the heart to increase its rate appropriately in response to an increased demand, is believed to be an important mechanism contributing to an impaired exercise functional capacity in HFpEF and may be made worse by BB [147]. In fact, BB withdrawal was associated with improved functional capacity of patients with HFpEF through an improved chronotropic response [148].

#### 1.14.2. Renin-Angiotensin-Aldosterone System (RAAS) Inhibitors

Compensatory activation of the renin-angiotensin-aldosterone system (RAAS) is a pathophysiologic characteristic shared across the HF spectrum [139, 140]. Angiotensin-converting enzyme inhibitors (ACEIs), angiotensin receptor antagonists (ARBs), and mineralocorticoid receptor antagonists (MRAs) inhibit components of the RAAS system. Angiotensin receptor neprilysin inhibitors (ARNI) combine the RAAS inhibition via an ARB (valsartan) with the inhibition of neprilysin (sacubitril). The additional inhibition of neprilysin increases levels of endogenous vasoactive peptides such as bradykinin and natriuretic peptides which help counteract RAAS effects. Clinical trials of RAAS inhibitors in HFpEF have produced mixed results.

#### 1.14.3. ACE Inhibitors (ACEI)

That have been no large (*n* > 1,000) clinical trials investigating ACEI in HFpEF. A meta-analysis of eight ACEI trials comprising just over 2,000 HFpEF patients found little or no effect on mortality, hospitalization, or quality of life [143].

#### 1.14.4. Angiotensin Receptor Blockers (ARBs)

The use of ARBs in HFpEF has been investigated in two large clinical trials: I-PRESERVE and CHARM-Preserved [149, 150]. Irbesartan, in the I-PRESERVE, failed to show a significant reduction in death or hospitalization [150]. The CHARM-Preserved investigated candesartan and similarly failed to find a significant difference in the primary composite outcome of CV death and HF hospitalization [149]. But there was a decreased in the risk of HF hospitalization based on a prespecified secondary analysis. A post hoc analysis of the CHARM-Programme, which included HF patients across the EF spectrum, found no significant statistical interaction between EF and candesartan effect [136]. Additionally, a spline regression analysis with EF as a continuous variable showed that candesartan may reduce CV death or HF hospitalization as EF goes beyond 50%; however, the efficacy declines as EF increases beyond 50%. However, a meta-analysis of ~8,000 HFpEF patients from 4 trials found no signal of benefit for ARBs on mortality, hospitalization, or quality of life [143].

#### 1.14.5. Angiotensin Receptor Neprilysin Inhibitor (ARNI)

In terms of treatment options with the angiotensin receptor neprilysin inhibitor (ARNI) sacubitril/valsartan, the PARAMOUNT (Prospective Comparison of Angiotensin Receptor Neprilysin Inhibitor with Angiotensin Receptor Blocker on Management of Heart Failure with Preserved Ejection Fraction) trial demonstrated that this ARNI resulted in greater reduction in N-terminal pro–B-type natriuretic peptide and reduction in LA size compared with valsartan [151].

Furthermore, the PARAGON-HF (Efficacy and Safety of the angiotensin receptor neprilysin inhibitor (ARNI) sacubitril/valsartan compared to Valsartan, on Morbidity and Mortality in Heart Failure Patients With Preserved Ejection Fraction) study assessed the efficacy of these interventions in patients with history of AF or documented AF at time of enrollment. Unfortunately, the presence of this arrhythmia is noted to be associated with a higher risk of total HF hospitalizations and CVD death [152]. However, first detection of AF was not influenced by use of sacubitril/valsartan [152].

Further data analysis later published by the PARAGON-HF investigators compared ARNI against ARB in over 10,000 patients with HF and an EF > 45% to determine the benefit regarding CV death and HF hospitalizations [153]. In this subsequent analysis, a signal of benefit for ARNI on CV death and HF hospitalization was suggested even though it did not achieve statistical significance (rate ratio, 0.87; 95% CI, 0.75-1.01; *P* = 0.06) [153]. Analyses of prespecified subgroups indicated a benefit in patients with an EF between 45 and 57%, those with a glomerular filtration rate (GFR) < 60 ml/min and in women [153].

Further analysis of the PARAGON-HF expanded the composite endpoint by including worsening HF events urgently treated in the ambulatory setting without hospitalization [154]. These urgent ambulatory visits for worsening HF were shown to be prognostically important in HFpEF, and the addition of the data reinforced the treatment efficacy of ARNI versus ARB alone (RR: 0.86; 95% CI, 0.75-0.99; *P* = 0.040) [154].

#### 1.14.6. Mineralocorticoid Receptor Antagonists (MRA)

Spironolactone, in the TOPCAT trial, failed to reduce all-cause mortality and HF hospitalizations in HFpEF (EF > 45%) patients [155]. However, there was a small, borderline significant improvement in rates of HF hospitalizations (hazard ratio, 0.83; 95% CI, 0.69 to 0.99, *P* = 0.04). Several secondary analyses have further assessed the efficacy of MRA in HFpEF. A post hoc analysis found a regional interaction where patients from the Americas, opposed to Russia/Georgia, randomized to spironolactone, had a significant reduction in the primary composite outcome of death and HF hospitalizations (hazard ratio, 0.82; 95% CI, 0.69–0.98) [156].

The relationship between EF (ranging from 44 to 85%) and the efficacy of spironolactone found that the potential benefit was strongest at the lower end of the EF spectrum [157]. A machine learning phenotypic analysis of clinically distinct subgroups found that spironolactone has a pronounced improvement in death and HF hospitalization in patient phenotype subgroup that demonstrated more functional impairment, obesity, diabetes, chronic kidney disease, concentric LV hypertrophy, high renin, and biomarkers of tumor necrosis factor-alpha-mediated inflammation, liver fibrosis, and tissue remodeling [158]. Spironolactone did not have a significant effect in the other phenogroups: (1) younger age, higher prevalence of smoking, preserved functional class, and the least evidence of left ventricular (LV) hypertrophy and arterial stiffness and (2) older age, with normotrophic concentric LV remodeling, atrial fibrillation, left atrial enlargement, large-artery stiffening, and biomarkers of innate immunity and vascular calcification.

#### 1.14.7. SGLT-2 Inhibition

The most promising pharmacotherapy for HFpEF, to date, is the sodium-glucose cotransport-2 inhibitors (SGLT2i). Having been shown to reduce the development and progression of HF in patients with type 2 diabetes and in those with HFrEF, the EMPEROR-PRESERVED trial sought to investigate the efficacy of the SGLT2i empagliflozin in HFpEF (EF > 40%) patients [159–161]. Those randomized to empagliflozin had a reduced risk of CV death or total HF hospitalization (hazard ratio, 0.79; 95% CI, 0.69 to 0.90; *P* < 0.001) [157]. The benefit was driven by a reduction in HF hospitalizations. A subgroup analysis by EF found that there was no statistically significant treatment interaction by EF subgroups (<50%, 50%–<60%, and >60%), but the benefits were attenuated at higher end of the EF spectrum [162].

The CHIEF-HF trial demonstrated that canagliflozin significantly improves symptom burden in HF within 12 weeks, regardless of EF [163]. Furthermore, in the PRESERVED-HF trial, dapagliflozin was found to significantly improve exercise function, physical limitations, and patient-reported symptoms at 12 weeks in HFpEF (EF > 45%) patients [164]. A randomized trial investigating the efficacy of dapagliflozin on HFpEF outcomes is currently underway [165].

Two trials have investigated SGLT2i use in patients recently hospitalized for HF irrespective of EF. In the SOLOIST-WHF trial, the nonselective SGLT1/2 inhibitor sotagliflozin significantly reduced the rate of CV death and HF hospitalizations in patients with diabetes. This effect was consistent across the prespecified subgroup stratified by EF < 50 or ≥50% [166]. The EMPULSE trial found that empagliflozin was safe in the acute HF setting and resulted in clinical benefit at 90 days. Clinical benefit primary endpoint was defined by a hierarchical composite of death from any cause, number of HF events and time to first HF event, or a 5 point or greater difference in change from baseline in the Kansas City Cardiomyopathy Questionnaire Total Symptom Score [116]. There was no statistical interaction between patients with a baseline EF above or below 40%.

The mechanism by which the SGLT2i provide protection in HF has not been fully elucidated.

## 2. Conclusions

Over the last two decades, the changing paradigm of HFpEF has become quite evident. In the late 1990s, HFpEF was not even a clinical term, and patients afflicted with similar symptoms were generally referred to as patients suffering from “diastolic heart failure.” This term used to encompass a group of patients mainly viewed as having small and thick left ventricles with abnormal diastolic filling patterns as their main pathophysiologic abnormality. However, emerging data has shown us that HFpEF is a more complex and dynamic clinical entity that embodies numerous mechanisms and is linked to multiorgan dysfunction, with hypertension and obesity playing a major role.

It should now be obvious why prior clinical trials on HFpEF have been for the most part frustrating when compared to HFrEF as HFpEF involves multiple pathophysiological mechanisms, which result in the heterogeneous phenotypes that have potentially confounded previous HFpEF trial results.

Even though we have gained an enormous amount of understanding not only on the causes but also downstream effects of HFpEF, there is still much to be learned before we can fully comprehend this complex clinical entity.

We hope that this review has synthesized what we know and which direction we should take.

## Figures and Tables

**Table 1:** Supplemental echocardiographic measures for LV diastole assessment.

Measurement	Significance	Utility
Mitral valve inflow deceleration time (DT)	Equated with LV chamber stiffness. Measure independent of heart rate, contractility, and afterload.	More sensitive and specific that E/A ratio. Recent cardioversion for atrial fibrillation. A short DT post myocardial infarction associated with worse prognosis.

Tricuspid Regurgitation	In up to 25% of patients with pulmonary arterial hypertension have occult LVDD and might require fluid challenge or exercise for proper documentation.	Pulmonary hypertension might be simply reflective of pulmonary parenchymal or vascular disease rather accompanying LVDD.

LV color-M-mode	Excellent spatiotemporal map of blood flow velocities along the scan line from the MA to the LV apex. Color M-mode correlates with the time constant of LV relaxation.	Measuring the slope of the isovelocity contour has been used to examine diastolic function based on how rapid (normal) or reduced (abnormal) this slope of flow moves away from MA. Different slope angulations can be obtained between the M-mode cursor and flow resulting in potential erroneous measurements that can compromise reproducibility and feasibility.

**Table 2:** Aging and the cardiovascular system.

Cardiovascular abnormalities	
Site	Main findings
Heart: structural changes	(i) Mild increase in heart weight (ii) Age-dependent change in cardiac shape (iii) Rightward shift in the ascending aorta (iv) Proximal bulge in the interventricular septum (v) Increased cardiomyocyte dimensions (vi) Decreased number of cardiomyocytes (vii) Quantitative and qualitative changes in collagen (viii) No increase in the collagen-to-myocyte ratio (ix) Partial degeneration of cardiac sympathetic nerve supply

Heart: functional changes	(i) In the resting aging heart, no alterations on LV systolic function as resting heart rate if anything minimally reduced with aging and cardiac output is preserved (ii) LV diastolic function undergoes significant age-related changes, with a reduction in early diastolic filling compensated for by increased end-diastolic filling and a consequent progressive reduction of the echocardiographic early wave/atrial wave (E/A) velocity ratio (iii) Both the catecholamine- or exercise-induced increases in heart rate and myocardial contractility are definitely blunted in elderly subjects (iv) Peak cardiac output attained in response to maximal effort is blunted by some 20–30% in elderly compared with young healthy subjects (v) Aging is accompanied by a blunted inotropic but preserved chronotropic response, and although LV filling reserve declines with age, relaxation reserve does not.

Vasculature: structural changes	(i) Large arteries are elongated and tortuous (ii) Enlarged lumen and a thickened walls (iii) Thickening mainly affecting the intima and the media (iv) Endothelial cells might become irregular in shape and have increased height (v) Migration and/or proliferation of vascular smooth muscle cells (vi) Exaggerated deposition of collagen, elastin, and proteoglycans, along with abnormal abundance of leukocytes and macrophages (vii) More abundant number of adhesion molecules, matrix metalloproteinases, transforming growth factor-*β*, and proinflammatory cytokines

Vasculature: functional changes	(i) Impaired distensibility (ii) Enhanced pulse wave velocity (iii) Increased stiffness (iv) Abnormal humoral and endothelial regulation of vascular smooth muscle tone (v) Increased endothelial permeability and a reduced nitric oxide-dependent vasodilator response to acetylcholine (vi) Moderate increase in total peripheral resistance (vii) Vessel wall hypertrophy

Arterial baroreflexes	(i) Age-related decline in the ability to modulate cardiac chronotropic activity (ii) Age-related depression of the baroreceptor-heart rate reflex (iii) Aging is associated with a blunted reflex changes in R-R interval in response to a change in BP (iv) Increased levels of oxidative stress and decreased cardiac cholinergic responsiveness with age (v) Increased levels of BP variability (vi) Impaired ability to respond to acute challenges to the maintenance of BP (vii) Increased risk of sudden cardiac death (viii) In contrast, baroreflex control of sympathetic outflow is not impaired with age

Cardiopulmonary reflexes	(i) Less attention given to these reflexes (ii) They control plasma renin activity and renal function (iii) Blunt hemodynamic and humoral component of the cardiopulmonary reflex with aging (iv) Preserved rather than attenuated cardiopulmonary reflex control of forearm vascular resistance

**Table 3:** Additional clinical entities useful in improving phenotypic characterization of HFpEF.

Variable	Abnormality	Functional correlate
Endothelial dysfunction	Increased circulating levels of IL-6 and TNF-*α*. Increased endothelial production of ROS.	Increased myocyte stiffness.

Reduced microvascular density	Microvascular rarefaction.	Increased myocardial fibrosis.

Peripheral vascular dysfunction	Increased central arterial stiffness and increased magnitude of arterial wave reflections.	Increased afterload. Increased LVH.

Impaired skeletal muscle vasodilatory reserve during exercise	Results in a blunted exercise-induced reduction in systemic vascular resistance and presumed abnormal skeletal muscle oxygen delivery.	Exercise intolerance.

Pulmonary hypertension	Due to pulmonary vascular remodeling secondary to sustained pulmonary venous pressure elevation, primary abnormalities in pulmonary arterial function, and abnormal right ventricle RV–PA coupling.	Exercise intolerance and dyspnea on exertion.

Lung disease	Airflow limitation	Exercise intolerance.

Obstructive sleep apnea	Impairs LV diastole	Begets LVH and may hasten HFpEF progression.

Chronic kidney disease	Adverse outcomes CKD is associated with worse outcomes in HFpEF rather in HFrEF	RV/LV remodeling and LV longitudinal systolic dysfunction. Poor diuretic response.

Atrial fibrillation	Increased LA stiffness and greater LA pulsatility	Associated with aging and results in more hospitalizations and poor prognosis independent of stroke risk

Frailty	Increased with unhealthy aging.	More comorbidities and associated with greater ED visits and hospitalizations.

Legend: IL=interleukin; TNF-*α*=tumor necrosis factor-alpha; ROS=reactive oxygen species; LVH=left ventricular hypertrophy; RV–PA=right ventricle-pulmonary artery; CKD=chronic kidney disease; and ED=emergency department.

**Table 4:** Table summarizing all the relevant clinical trials for the different classes of drugs in this review with regards to HFpEF.

Clinical trial	Class of medication	Summary
OPTIMIZE-HF registry	Beta-blockers	In patients with HFpEF and heart rate ≥ 70 beats per minute, high-dose beta-blocker use was associated with a significantly lower risk of death [146].

CHARM-Preserved	Angiotensin receptor blockers	Candesartan has a moderate impact in preventing admissions for CHF among patients who have heart failure and LVEF higher than 40% [149].

I-PRESERVE	Angiotensin receptor blockers	Irbesartan did not improve the outcomes of patients with heart failure and a preserved left ventricular ejection fraction [150].

PARAGON-HF	Angiotensin receptor neprilysin inhibitor against angiotensin receptor blockers	Sacubitril-valsartan did not result in a significantly lower rate of total hospitalizations for heart failure and death from cardiovascular causes among patients with heart failure and an ejection fraction of 45% or higher [151].

TOPCAT trial	Mineralocorticoid receptor antagonists	In patients with heart failure and a preserved ejection fraction, treatment with spironolactone did not significantly reduce the incidence of the primary composite outcome of death from cardiovascular causes, aborted cardiac arrest, or hospitalization for the management of heart failure [153].

EMPEROR-PRESERVED trial	SGLT-2 inhibition	Empagliflozin reduced the combined risk of cardiovascular death or hospitalization for heart failure in patients with heart failure and a preserved ejection fraction, regardless of the presence or absence of diabetes [159].

CHIEF-HF trial	SGLT-2 inhibition	Canagliflozin significantly improves symptom burden in heart failure within 12 weeks, regardless of ejection fraction [161].

PRESERVED-HF trial	SGLT-2 inhibition	Dapagliflozin was found to significantly improve exercise function, physical limitations, and patient-reported symptoms at 12 weeks in HFpEF (EF > 45%) patients [162].

SOLOIST-WHF trial	Nonselective SGLT1/2 inhibitor	Sotagliflozin significantly reduced the rate of CV death and HF hospitalizations in patients with diabetes [164].

EMPULSE trial	SGLT-2 inhibition	Empagliflozin was safe in the acute heart failure setting and resulted in clinical benefit of death from any cause, number of heart failure events and time to first heart failure event, or a 5 point or greater difference in change from baseline in the Kansas City Cardiomyopathy Questionnaire Total Symptom Score at 90 days [165].
